# Nucleobindin 1 binds to multiple types of pre-fibrillar amyloid and inhibits fibrillization

**DOI:** 10.1038/srep42880

**Published:** 2017-02-21

**Authors:** Alessandra Bonito-Oliva, Shahar Barbash, Thomas P. Sakmar, W Vallen Graham

**Affiliations:** 1 Laboratory of Chemical Biology & Signal Transduction, The Rockefeller University, New York, NY 10065, USA; 2 Department of Neurobiology, Care Sciences and Society, Center for Alzheimer Research, Division of Neurogeriatrics, Karolinska Institutet, 141 57 Huddinge, Sweden

## Abstract

During amyloid fibril formation, amyloidogenic polypeptides misfold and self assemble into soluble pre-fibrillar aggregates, *i.e.*, protofibrils, which elongate and mature into insoluble fibrillar aggregates. An emerging class of chaperones, chaperone-like amyloid binding proteins (CLABPs), has been shown to interfere with aggregation of particular misfolded amyloid peptides or proteins. We have discovered that the calcium-binding protein nuclebindin-1 (NUCB1) is a novel CLABP. We show that NUCB1 inhibits aggregation of islet-amyloid polypeptide associated with type 2 diabetes mellitus, a-synuclein associated with Parkinson’s disease, transthyretin V30M mutant associated with familial amyloid polyneuropathy, and Aβ42 associated with Alzheimer’s disease by stabilizing their respective protofibril intermediates. Kinetic studies employing the modeling software AmyloFit show that NUCB1 affects both primary nucleation and secondary nucleation. We hypothesize that NUCB1 binds to the common cross-β-sheet structure of protofibril aggregates to “cap” and stabilize soluble macromolecular complexes. Transmission electron microscopy and atomic force microscopy were employed to characterize the size, shape and volume distribution of multiple sources of NUCB1-capped protofibrils. Interestingly, NUCB1 prevents Aβ42 protofibril toxicity in a cellular assay. NUCB1-stabilized amyloid protofibrils could be used as immunogens to prepare conformation-specific antibodies and as novel tools to develop screens for anti-protofibril diagnostics and therapeutics.

Amyloidogenic polypeptides have the capacity to form a characteristic cross-β-sheet structure and progressively self-assemble into soluble oligomers and protofibrils, which are large heterogeneous aggregates of up to several hundred polypeptides. As the protofibrils extend and “mature,” they become insoluble and form Congo red-positive deposits, often called amyloid plaques. Amyloid aggregates present in the brain are associated with a reduction in the efficiency of coordinated synaptic transmission, loss of synaptic plasticity and contribute to cognitive impairment in many diseases such as Alzheimer’s disease (AD)^1^, Parkinson’s disease (PD)^2^,^3^, Huntington’s disease (HD)^4^ and frontotemporal lobar degeneration (frontotemporal dementia, clinical amyotrophic lateral sclerosis (ALS), and motor neuron disease)^5^,^6^. Aggregation of misfolded proteins in the periphery of the body also results in amyloidosis syndromes including diabetes mellitus (DM) type 2^7^, familial amyloid cardiomyopathy (FAC), familial amyloid polyneuropathy (FAP)^8^, and systemic light-chain (AL) amyloidosis^9^,^10^.

There are at least 31 proteins and peptides in humans that are classified as amyloidogenic because the tissue deposits they form exhibit birefringence under polarizing light when stained with Congo red^11^. These peptides and proteins lack significant primary structure homology, although they form aggregates that share the common cross-β-sheet structure^12^,^13^. Increasing evidence suggests that a common transient conformation, rather than the specific amino acid sequence, underlies a universal pathogenic mechanism for different amyloid proteins^13^,^14^. For example, AD is a neurodegenerative disorder histopathologically characterized by amyloid-β (Aβ) deposits, neurofibrillary tangles of tau and dystrophic neurites^15^,^16^. The amyloid-forming Aβ peptide is a proteolytic fragment of the transmembrane Amyloid Precursor Protein (APP)^17^ generated from sequential cleavage by β- and γ-secretases^18^. The classic hypothesis of extracellular Aβ plaques as the main toxic cause of the disease^19^,^20^ has been challenged by data suggesting that the soluble intermediate oligomers and protofibrils that precede Aβ plaque formation are the primary toxic entities and better correlate with cognitive impairment^13^,^14^,^21^,^22^.

The increasingly recognized pathogenic role of the intermediate protofibrils highlights the need for therapeutic approaches that prevent protofibril formation in order to suppress cellular toxicity^17^,^23^. Recent *in vitro* and *in vivo* studies have shown that molecular chaperones, or chaperone-like amyloid binding proteins (CLABPs), can efficiently inhibit amyloid formation and, therefore, might protect against amyloid-induced toxicity^24^,^25^,^26^,^27^,^28^,^29^,^30^,^31^,^32^. However, their influence on the kinetics of amyloid aggregation intrinsically depends on the type of amyloid polypeptide and the specific inhibited step in the aggregation process. In addition, the non-linear aggregation kinetics of amyloidogenic polypeptides presents a major challenge in characterizing the interaction of chaperones or amyloid-binding proteins with targets of interest.

Aβ is perhaps the best-described kinetic model of amyloid aggregation for understanding the pathways affected by CLABPs^33^. CLABPs that inhibit Aβ aggregation have been described to prevent microscopic events, such as primary nucleation, fibril elongation, fibril fragmentation, or secondary nucleation^28^,^30^,^34^. The molecular pathway that drives Aβ amyloid formation is predominantly through fibril-catalysed secondary nucleation events^33^. Recently, proposed models that predict the interaction between molecular chaperones and various amyloids set a platform for the investigation of their modulating effects on misfolded protein aggregation^31^,^35^. The inhibitory effect of CLABPs on amyloid aggregation can be modelled based on the specific and predictable changes in the rate constants of aggregation. For example, the extracellular chaperone clusterin, as well as several heat shock proteins (HSPs) (*i.e.*, HSP70 and HSP90), have been shown to inhibit aggregation of amyloidogenic peptides by binding to, and stabilizing, prefibrillar species^36^,^37^,^38^,^39^. Whereas DNAJB6 prevents Aβ aggregation by mainly acting on primary nucleation pathways^40^, the molecular chaperone domain BRICHOS inhibits *in vitro* Aβ aggregation by interfering potently and selectively with the secondary nucleation reaction^18^,^31^,^34^,^41^.

Nucleobindin 1 (NUCB1) is a 55-kDa multi-domain Golgi-resident Ca^2+^-binding protein that has been shown to be “membrane active” and can bind heterotrimeric guanine-nucleotide binding proteins^42^,^43^,^44^. In the Golgi, NUCB1 plays an important role in modulating Ca^2+^ homeostasis and is a negative regulator of the unfolded protein response through inhibition of site-1 protease (S1P)- mediated cleavage of ATF6^45^,^46^,^47^.

While NUCB1 has not been directly linked to human disease, up-regulation of the gene has been found in animal models of Lupus^48^,^49^. In post-mortem brains of AD patients, NUCB1 protein levels have been found to be reduced by an average of 50% compared with controls^50^,^51^. *In vitro* studies have shown that NUCB1 directly interacts with APP in a Ca^2+^-sensitive manner and its *in vitro* over-expression reduces the APP levels^50^. The Ca^2+^-dependent effect is particularly interesting in light of the dysregulated Ca^2+^ homeostasis shown in AD pathology^52^,^53^,^54^ as well as in other neurodegenerative diseases, such as PD, HD, familial amyloidosis syndromes and ALS^55^. We have previously shown that an engineered form of NUCB1 (*s*NUCB1) prevents *in vitro* aggregation of the human amyloid polypeptide (hIAPP) whose aggregation is associated to type 2 DM^44^. We found that, in this model, *s*NUCB1 binds to prefibrillar species and prevents hIAPP-induced cytotoxicity^44^. However, these effects were prevented in presence of Ca^2+^ 44 making *s*NUCB1 an unstable tool for use in *in vivo* models.

## Results and Discussion

To study the amyloid binding capability of NUCB1 in the presence of Ca^2+^, we engineered a mutant variant of *s*NUCB1 (*mt*NUCB1) that is unable to bind Ca^2+^ but retains its hIAPP-binding activity. The Ca^2+^-free *mt*NUCB1 failed to bind Ca^2+^ as judged by isothermal titration calorimetry (ITC) (data not shown), in spite of a preserved structure characteristic of a fully folded protein with a predominant α-helical secondary structure, as measured by circular dichroism (CD) (Supplementary Fig. S1). Thermal denaturation experiments revealed an unfolding transition with an apparent Tm of 48.7 °C (Supplementary Fig. S1), similar to that of *s*NUCB1^44^. Atomic Force Microscopy (AFM) experiments show that *mt*NUCB1 is present as both monomers and dimers with asymmetric and heterogeneous shape and height up to 1.4 nm (Supplementary Fig. S1). However, in line with previous reports^43^, the AFM volume analysis suggests that the protein has a predominant dimeric conformation, as indicated by a larger peak at ~88 nm^3^ and a smaller peak at ~44 nm^3^ (Supplementary Fig. S1), and further confirmed by gel analysis (data not shown). Finally, the biophysical analysis of these data not only revealed that *mt*NUCB1 monomers and dimers have different shape distributions, but also indicated a more heterogeneous dimer population (Supplementary Fig. S1).

We first observed that Aβ42 aggregation, measured under quiescent conditions by thioflavin-T (Thio-T) fluorescence depends on the monomeric concentration of the peptide in solution (Supplementary Fig. S2), in line with the previously described rate constants of Aβ42 aggregation^33^. The time at which half the protein initially present in soluble form has aggregated (half-time) is a valid gauge to investigate the dependence of the aggregation on the initial monomeric concentration. The half-times for increasing Aβ42 concentrations were calculated with the online software AmyloFit (http://www.amylofit.ch.cam.ac.uk) and graphed in a double logarithmic plot, where the resulting slope gives the scaling exponent that can be used to study the reaction’s molecular mechanism. We found that, in absence of *mt*NUCB1, the scaling exponent calculated across Aβ42 concentrations was −0.711, slightly higher than previous reports with recombinant Aβ42^33^ or depsipeptide- derived sources^39^, and it remained relatively stable over the range of examined concentrations (Supplementary Fig. S2). These data are indicative of a secondary nucleation dominant Aβ42 aggregation, in agreement with Cohen *et al*.^33^.

To further characterize Aβ42 aggregation, we performed transmission electron microscopy (EM) experiments and observed that after 24 h of incubation at 37 °C the complexity and size of the aggregates increase with increasing monomeric concentrations (Supplementary Fig. S2), whereas the classic twisting structure^56^ was observed under all conditions tested (not shown). The description of the Aβ42 aggregation kinetics is further complemented by immunoelectron microscopy (immunoEM) experiments showing anti-Aβ antibody-positive early aggregates, protofibrils and fibrils appearing after 20 min, 1 h or 24 h, respectively, of incubation at 37 °C (Supplementary Fig. S2).

To test the hypothesis that engineered Ca^2+^-free *mt*NUCB1 has an effect on *in vitro* aggregation of Aβ42, we performed two sets of experiments testing the inhibitory effect of *mt*NUCB1 on low (2.5 μM) (A-D) and high (10 μM) (E-H) Aβ42 concentrations. These experiments were designed in order to manipulate Aβ42 aggregation speed while comparing similar *mt*NUCB1: Aβ42 ratios. We show that *mt*NUCB1 inhibits aggregation of both 2.5 μM and 10 μM Aβ42 concentrations (Supplementary Fig S3) and reduces fibril mass in a dose-dependent manner, completely inhibiting measurable aggregation when *mt*NUCB1 is 1.6x or 4x the molar concentration of low- and high-concentration Aβ42, respectively (Supplementary Fig. S3). Interestingly, 40 μM *mt*NUCB1 is able to completely inhibit 10 μM Aβ42 aggregation for up to 72 h (data not shown).

**Figure 1:**
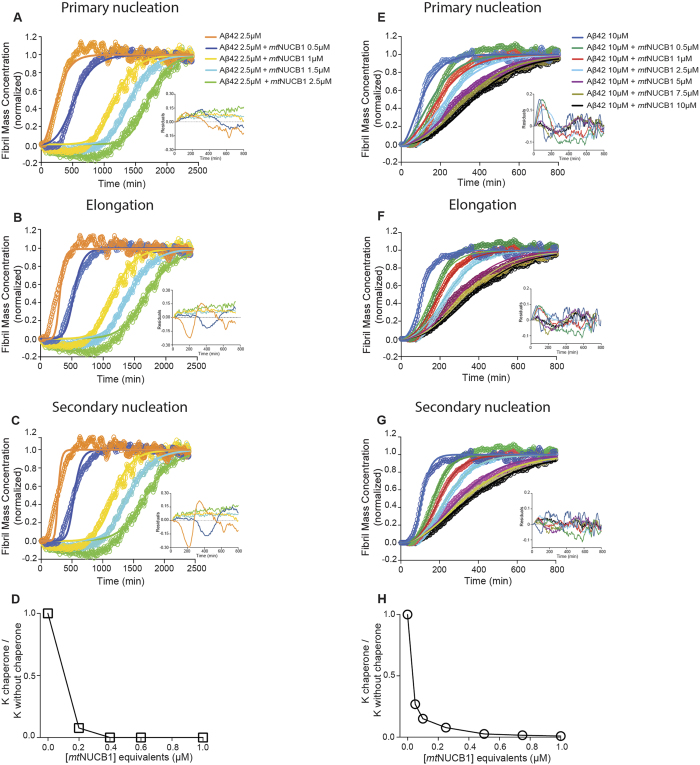
Kinetic analysis of *mt*NUCB1 inhibition of aggregation mechanisms. (**A–D**) Aggregation of 2.5 μM Aβ42 and (**E–G**) 10 μM Aβ42 in presence of different *mt*NUCB1 concentrations was measured by Thio-T assay and analysed by AmyloFit. (**A**,**E**) For each Aβ42 concentration, the aggregation plateau values were normalized to 1 and the rate constant parameters for primary nucleation, (**B**,**F**) elongation and (**C**,**G**) secondary nucleation were individually fitted in parallel to global fitting of the other two, and the fitted (lines) and the experimental (circles) data were compared. In each panel, the insets show the fitting residuals over time obtained from the respective analysis. (**D**) Fitted rate constants for secondary and primary nucleation are shown for low and (**H**) high Aβ42 concentrations, respectively, as a function of *mt*NUCB1 equivalent to Aβ42 concentration. The rate constants are normalized relative to the values in the absence of *mt*NUCB1.

The inhibitory effect of *mt*NUCB1 on Aβ42 aggregation was thoroughly investigated through AmyloFit to understand the relative contribution of *mt*NUCB1 to each microscopic aggregation event (Fig. 1). First, for each of the two sets of experiments, all the kinetic constants (primary nucleation, elongation and secondary nucleation) were individually analyzed and discrete simulations were performed by changing only one parameter at a time and globally fitting the remaining two (Supplementary Table S1 and Supplementary Table S2). The analysis suggests that, in the presence of low (2.5 μM) Aβ42 concentration, *mt*NUCB1 mainly affects primary nucleation. The best fit of the data to the model of inhibition of primary nucleation is indicated by the smallest mean residual error (MRE) (primary nucleation = 0.0038, elongation = 0.0040, secondary nucleation = 0.0049) and the reduced dispersion of the corresponding residuals over time (Fig. 1A–C). Interestingly, in the presence of high (10 μM) Aβ42 concentration, *mt*NUCB1 acts by mainly inhibiting secondary nucleation. In this case, the best fit of the data is to the model of inhibition of secondary nucleation as shown by the smallest MRE (secondary nucleation = 0.0009, elongation = 0.0013, primary nucleation = 0.0019) and the reduced dispersion in the corresponding residuals (Fig. 1E–G). Notably, the *mt*NUCB1: Aβ42 ratio does not change between the two experiments and it is therefore reasonable to speculate that the slower aggregation occurring in the low Aβ42 concentration sample unmasks the ability of *mt*NUCB1 to interfere with early microscopic aggregation events. On the other hand, in the case of a rapid aggregation occurring in the high Aβ42 concentration sample, *mt*NUCB1 is likely to have a more complex inhibitory effect, possibly binding to long fibrils to prevent secondary nucleation. Taken together, our analyses support the hypothesis that *mt*NUCB1 prevents Aβ42 fibril formation through a complex dual mechanism that is ratio-dependent.

The inhibition of primary nucleation disrupts the fibrillization of early aggregates, whereas the inhibition of secondary nucleation occurs when an inhibitor prevents the catalysation of aggregation at the fibril surface. A mechanism of *mt*NUCB1 inhibiting two kinetic features of Aβ42 aggregation would result in a variable ratio between monomers, short aggregates and long fibrils over a series of *mt*NUCB1 concentrations. In light of the linear decrease in Aβ42 fibril mass with increasing concentrations of *mt*NUCB1 (Supplementary Fig. S3), and according to the proposed ratio-dependent mechanism of *mt*NUCB1 inhibition, it is reasonable to expect non-linear changes in the concentration of the reaction products, *i.e.*, monomers and protofibrils. Specifically, when the inhibition occurs through secondary nucleation, an increase of *mt*NUCB1 concentration would result in a progressive lack of monomer conversion to fibril. On the other hand, *mt*NUCB1 inhibition of early aggregation steps may result in an increase of Thio-T negative intermediate aggregates.

**Figure 2:**
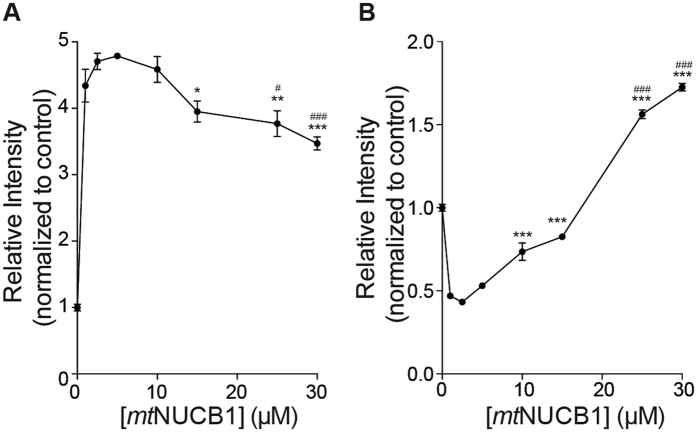
The *mt*NUCB1: Aβ42 ratio determines Aβ42 monomer and soluble aggregates concentration. Samples containing 10 μM Aβ42 and 0, 1, 2.5, 5, 10, 15, 25, and 30 μM *mt*NUCB1 were incubated for 24 h at 37 °C in quiescent conditions. (**A**) Quantification of monomer concentration. At the end of the reaction, the monomer concentration was measured. Samples were ultracentrifuged and the supernatant fraction was separated with SEC. Eluted fractions of monomer were pooled and the Aβ42 content was measured by indirect ELISA. (**B**) Quantification of soluble aggregates concentration. A portion of the supernatant sample was probed with indirect ELISA using the anti-oligomer antibody A11 to detect small soluble aggregates. **p < 0.01, ***p < 0.001 *vs* 5 μM *mt*NUCB1. ^#^p < 0.05, ^###^p < 0.001 *vs* 10 μM. One-way ANOVA followed by Tukey’s post-hoc comparison.

To test these hypotheses, we determined monomer concentration in the presence of 10 μM Aβ42 and increasing concentrations of *mt*NUCB1 through size exclusion chromatography (SEC) and ELISA (Fig. 2A), dot blot and Western blot (not shown). The results show a monomer concentration increase from 0 to 5 μM *mt*NUCB1 followed by a decrease to 30 μM *mt*NUCB1. Low *mt*NUCB1: Aβ42 ratios (1:10, 1:4, 1:2) result in a progressive increase in monomer concentration, presumably through *mt*NUCB1 binding to the fibril surface and inhibiting secondary nucleation, that ultimately prevents monomer conversion to fibril material. ImmunoEM data support this scenario and indicate that in a *mt*NUCB1: Aβ42 1:4 ratio sample, *mt*NUCB1 binds along the fibrils (data not shown). On the contrary, high *mt*NUCB1: Aβ42 ratios (1:1, 1.5:1, 2.5:1, 3:1) lead to a progressive decrease in monomer concentration due to a main *mt*NUCB1 inhibition of primary nucleation, likely through *mt*NUCB1 binding to and stabilizing early aggregates. In agreement with the latter scenario, double immunoEM experiments indicate that increasing concentrations of *mt*NUCB1 result in progressively decreased fibril mass (data not shown), in agreement with Thio-T data (Supplementary Fig. S3).

Our hypothesis is further supported by parallel experiments where we measured the soluble aggregate content present in the solutions at the end of the co-incubation of 10 μM Aβ42 and increasing concentrations of *mt*NUCB1 (Fig. 2B). The ELISA performed with the anti-oligomer A11 antibody indicates that increasing *mt*NUCB1 concentrations lead to an increased concentration of soluble aggregates from 1 to 30 μM *mt*NUCB1, with an inflection point around 2.5 μM *mt*NUCB1. The decrease observed in soluble aggregate content between 0 and 1 μM *mt*NUCB1 is in line with the hypothesis that at low *mt*NUCB1: Aβ42 ratio (1:10) *mt*NUCB1 mainly coats the fibril surface and inhibits secondary nucleation and surface catalysed aggregate seeds. This mechanism results in increased monomer (Fig. 2A) and decreased soluble aggregate (Fig. 2B) concentration.

Altogether these data represent a clear indication that, in presence of *mt*NUCB1, the conversion of Aβ42 monomers into fibrils is incomplete. In fact, increasing concentrations of our CLABP result in a linear and progressive decrease in fibril mass (Supplementary Fig. S3), complex inverted U-shaped curve in monomer content and progressive non-linear increase in soluble aggregates concentration (Fig. 2).

**Figure 3:**
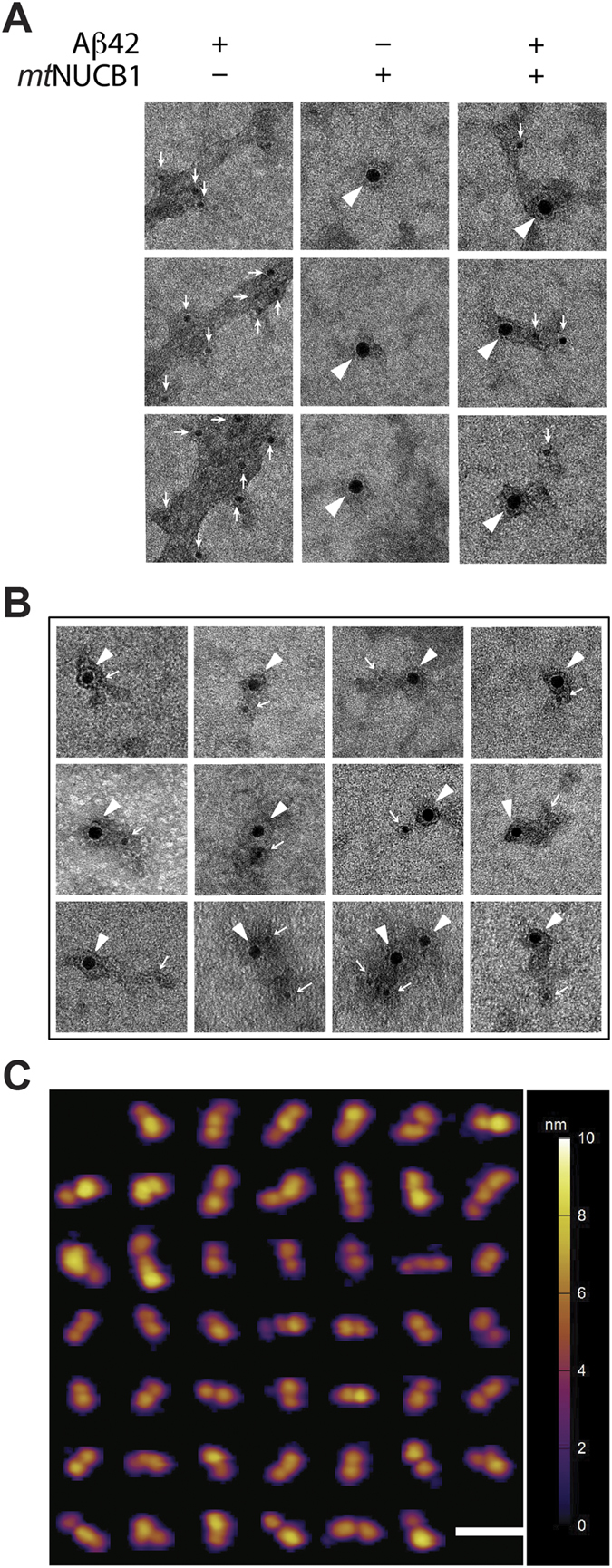
*mt*NUCB1 stabilizes short Aβ42 protofibrils. (**A**) Representative EM images of 10 μM Aβ42, 10 μM *mt*NUCB1, and 10 μM Aβ42+10 μM *mt*NUCB1 incubated at 37 °C for 24 h. All samples were incubated with both mouse anti-Aβ 6E10 and rabbit anti-NUCB1 antibodies and successively with the 6-nm gold-conjugated anti-mouse antibody and 12-nm gold-conjugated anti-rabbit antibody. Panel shows three 100 × 100 nm images per group. Arrowheads indicate 12-nm gold particles (*mt*NUCB1) and small arrows indicate 6-nm gold particles (Aβ42). (**B**) Representative EM images of *mt*NUCB1-Aβ42 protofibrils purified with SEC and incubated with both mouse anti-Aβ 6E10 and rabbit anti-NUCB1 antibodies and successively with the 6-nm gold-conjugated anti-mouse antibody and the 12-nm gold-conjugated anti-rabbit antibody. The panel shows twelve 100 × 100 nm images with arrowheads indicating 12-nm gold particles (*mt*NUCB1) and small arrows indicating 6-nm gold particles (Aβ42). (**C**) Composite of representative *mt*NUCB1-Aβ42 protofibrils (n = 47) selected based on the volumetric analysis of the sample imaged by AFM. Integrated xy scale bar is 40 nm; the colorimetric scale bar indicates the height of the particles.

In agreement with the increased protofibril content with increasing *mt*NUCB1 concentrations, co-incubation of 10 μM *mt*NUCB1 and 10 μM Aβ42 results in short protofibrils (~80 nm length and ~10 nm wide) that are positive for both the anti-NUCB1 and anti-Aβ antibodies (Fig. 3A, right column). Taken together, these data support the hypothesis that *mt*NUCB1 can prevent aggregation by binding to both short species (inhibition of primary nucleation) and long fibrils (inhibition of secondary nucleation).

To characterize *mt*NUCB1-stabilized Aβ42 soluble protofibrils, 10 μM *mt*NUCB1 and 10 μM Aβ42 were then co-incubated and the sample was purified by SEC. Indirect ELISA indicates that the *mt*NUCB1-Aβ42 complex contains both *mt*NUCB1 and Aβ42 (Supplementary Fig. S4). Further analysis by double immunoEM confirms that the purified material is positive for both anti-NUCB1 and anti-Aβ antibody staining (Fig. 3B). AFM analysis performed on the SEC-purified *mt*NUCB1-Aβ42 complex shows the presence of protofibrils with height of 3.79 +/− 0.07 nm and length of 30.94 +/− 0.65 nm (Fig. 3C). The biophysical analysis of the shape distribution determined through Fourier transform indicates that the heterogeneity of protofibril shape complexity is less than that of *mt*NUCB1 (Supplementary Fig. S4). These data are also in line with Dynamic Light Scattering (DLS) results indicating that the *mt*NUCB1-Aβ42 complex has a polydisperse size population with an average hydrodynamic radius that is bigger than *mt*NUCB1 alone (6.16 +/− 0.06 nm *versus* 5.64 +/− 0.08 nm, respectively) (Supplementary Fig. S4).

Our data show that the engineered form of Ca^2+^-free *mt*NUCB1 affects the kinetics of Aβ42 fibril formation and efficiently inhibits Aβ42 *in vitro* aggregation through interaction with early aggregates, stabilization of short protofibrils, and preventing further fibrillization. We previously showed that *s*NUCB1 prevents hIAPP aggregation through protofibril binding and stabilization^44^. When taken together, these observations suggest that *mt*NUCB1 is a CLABP with protofibril binding activity and mechanisms of action that target concomitantly two mechanisms of amyloid aggregation. To further validate the CLABP activity of *mt*NUCB1, we tested its effect on the aggregation of other amyloid proteins.

**Figure 4:**
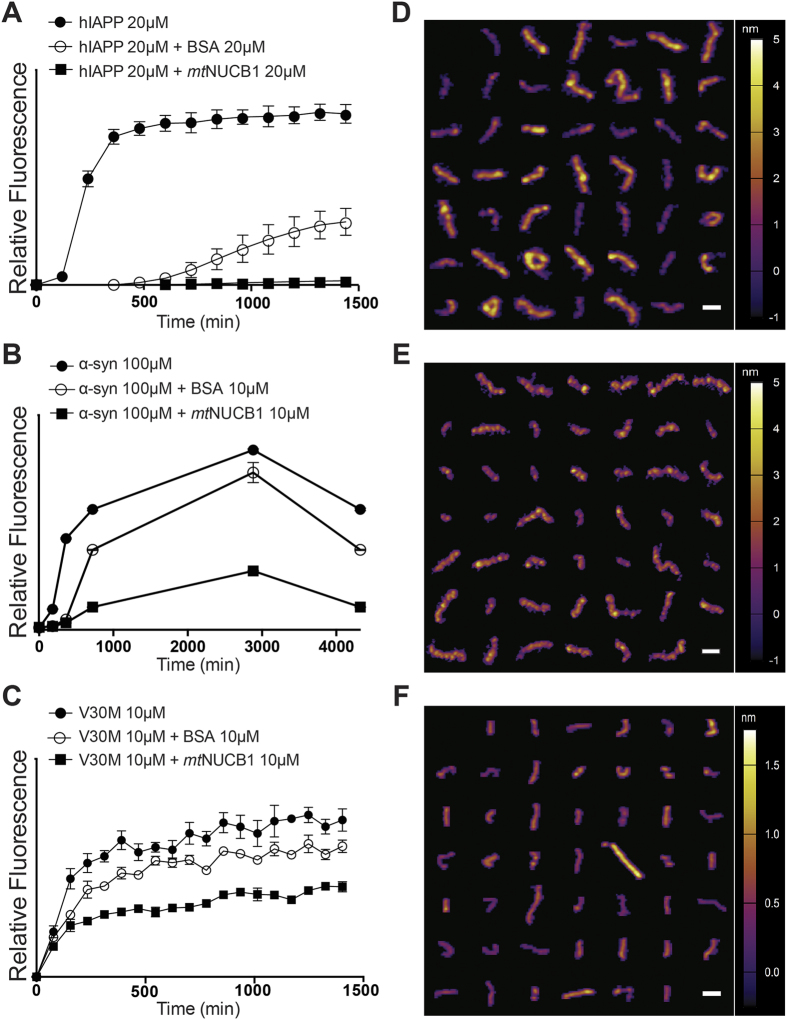
*mt*NUCB1 has pan amyloid chaperone-like activity. (**A**) hIAPP (30 μM) kinetics of aggregation was tested in absence or in presence of equimolar concentrations of *mt*NUCB1, or the control protein BSA, and 10 μM Thio-T at 25 °C in quiescent conditions, over 24 h. (**B**) α-synuclein (100 μM) aggregation was measured as end point fluorescence during incubation in absence or in presence of 10 μM *mt*NUCB1 or BSA, and 10 μM Thio-T, at 37 °C in shaking conditions, for 3 days. (**C**) The transthyretin V30M mutant (10 μM) aggregation was tested in absence or in presence of equimolar concentrations of *mt*NUCB1 or BSA, and 10 μM Thio-T, at 37 °C in quiescent conditions, over 24 h. (**D**) Composite of representative *mt*NUCB1- hIAPP, (**E**) *mt*NUCB1-α-synuclein and (**F**) *mt*NUCB1-V30M protofibrils purified by SEC and imaged by AFM. For each amyloid, representative protofibrils were selected based on the sample distribution of volumes. Integrated xy scale bar is 40 nm; colorimetric scale bar indicates the height of the particles.

We observed that *mt*NUCB1 not only inhibits aggregation of Aβ42, but also the type 2 DM associated hIAPP^7^ (Fig. 4A), α-synuclein associated with PD^57^ (Fig. 4B) and the transthyretin V30M mutant associated with FAP^8^ (Fig. 4C). The co-incubation of these different peptides with *mt*NUCB1 results in short protofibrils that can be isolated by SEC and characterized. The AFM analysis of these SEC-purified samples indicates that the *mt*NUCB1 binding to intermediate aggregates stabilizes protofibrils of different sizes and morphologies (Fig. 4D–F), depending on the amyloid protein. Indeed, *mt*NUCB1-Aβ42 protofibrils (Fig. 3C) are short (~30 nm long) and thick (~3.8 nm tall), as compared with the *mt*NUCB1-V30M protofibrils (Fig. 4F), which are slightly longer (64.36 +/− 2.76 nm) but thinner (0.9475 +/− 0.05 nm tall). In contrast, *mt*NUCB1-hIAPP (Fig. 4D) and *mt*NUCB1-α-synuclein (Fig. 4E) protofibrils have similar size (76.39 +/− 2.22 nm long and 3.41 +/− 0.14 nm tall, and 63.95 +/− 3.05 nm long and 3.40 +/− 0.07 nm tall, respectively), but display very different structures. Each of these protofibril samples consists of a population of species with elongated shape, but heterogeneous size. Distinct characteristics of the protofibrils were also elucidated through a volumetric analysis. *mt*NUCB1-Aβ42 and *mt*NUCB1-V30M protofibrils showed a similar distribution of volume with narrow peaks at ~200 nm^3^ and ~250 nm^3^, respectively, but completely different morphologies (Supplementary Fig. S5). *mt*NUCB1-hIAPP and *mt*NUCB1-α-synuclein protofibrils showed a more spread volume distributions with larger peaks at ~550 nm^3^ and ~600 nm^3^, respectively, and heterogeneous protofibril populations (Supplementary Fig. S5).

**Figure 5:**
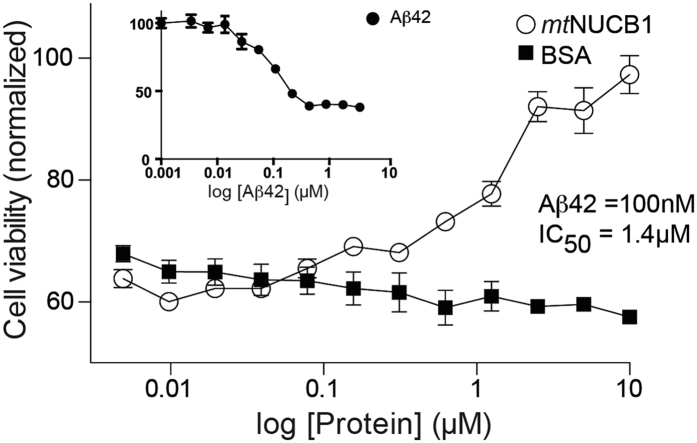
*mt*NUCB1 protects against Aβ42-induced toxicity. (**A**) Dose-dependent protective effect of *mt*NUCB1 against Aβ42-induced cell toxicity. PC12 cells were treated with 100 nM Aβ42 in the presence of *mt*NUCB1 or the control protein BSA. The cell viability was tested by MTT assay and indicates that *mt*NUCB1 has an IC_50_ of 1.4 μM. Inset shows the dose-dependent toxicity experienced by PC12 cells to Aβ42 as measured in an MTT cell viability assay and indicates an EC_50_ of 100 nM.

Finally, to determine whether *mt*NUCB1-stabilized Aβ42 protofibrils retain cytotoxic features, we first determined that 100 nM Aβ42 is the effective concentration to cause toxicity in 50% of PC12 cells in 24 h (Fig. 5, inset). We then evaluated the toxicity of Aβ42 in the presence of increasing doses of *mt*NUCB1 (Fig. 5). The results show that *mt*NUCB1 prevents 100 nM Aβ42 cytotoxicity with an IC_50_ of 1.4 μM. *mt*NUCB1 is protective against Aβ42-induced cytotoxicity and, therefore, stabilizes a nontoxic state of Aβ42 protofibrils. In light of the high toxicity exerted by intermediate, protofibril-like material^13^,^14^,^21^,^22^, the finding that the *mt*NUCB1 binding detoxifies Aβ42 protofibrils is extremely interesting. The mechanisms responsible for the Aβ42 protofibrils toxicity are still largely unknown, but our data suggest that *mt*NUCB1 may mask or otherwise act on the toxic features. More studies are needed to understand the underlying mechanism.

## Conclusions

Here we show that the CLABP *mt*NUCB1 can prevent aggregation of different amyloid proteins (*i.e.*, Aβ42, hIAPP, α-Syn, and transthyretin V30M) likely by binding to the common cross-β-sheet structure and stabilizing short, nontoxic protofibrils.

A detailed study on the inhibition of Aβ42 aggregation has highlighted a complex *mt*NUCB1 mechanism of action. First, we manipulated the speed of aggregation and showed that in the case of slow Aβ42 fibrillization (low Aβ42 concentrations) *mt*NUCB1 mainly affects initial aggregation steps by inhibiting primary nucleation. On the contrary, in the case of fast aggregation (high Aβ42 concentrations), *mt*NUCB1 seems to predominantly affect later aggregation steps by inhibiting secondary nucleation.

Successively, we manipulated the *mt*NUCB1: Aβ42 ratio and observed that in presence of low ratios (1:10, 1:4, 1:2) the reaction primarily relies on inhibition of secondary nucleation where *mt*NUCB1 coats the fibril surface resulting in a small decrease in fibril and soluble aggregate species but a large increase in free monomers. On the other hand, in case of high *mt*NUCB1: Aβ42 ratios (1:1, 1.5:1, 2.5:1, 3:1) *mt*NUCB1 binds to earlier aggregation species and inhibits primary nucleation resulting in a decrease in fibril and monomeric species and an increase in soluble aggregates.

Altogether these data indicate a complex dual *mt*NUCB1 mechanism of action, where the speed of the amyloid aggregation and the CLABP: amyloid ratio favours the inhibition of either primary or secondary nucleation.

Importantly, we show that in presence of *mt*NUCB1, the conversion of Aβ42 monomers into fibrils is incomplete and results in non-linear changes in the concentration of species other than fibrils, *i.e.*, monomers and protofibrils. In light of such a scenario, the models implemented in AmyloFit might not be ideal because they assume complete conversion of monomers. This work highlights the need for the refinement of existing tools for modeling complex molecular inhibitors of amyloid protein aggregation.

Since amyloid intermediates have increased cytotoxicity compared with mature fibrils^12^, there is an urgent need to develop tools to analyse, or therapeutics to prevent amyloid-induced toxicity. Recent structural studies provide new possibilities for understanding the interaction of *mt*NUCB1 with Aβ^58^,^59^. Our data indicate that the *mt*NUCB1 inhibition of Aβ42 aggregation is accompanied by prevention of intrinsic toxicity. Moreover, the finding that nontoxic *mt*NUCB1-Aβ42 complexes retain the structural properties of transient protofibrils and that these protein-amyloid complexes can be stabilized and isolated opens a range of possibilities for tool development. For example, we suggest that *mt*NUCB1 can be used to prepare stable, nontoxic protofibril immunogen for discovering conformation-specific, anti-protofibril antibodies, or for screening assays to develop small molecule inhibitors of amyloid fibrillization.

## Material and Methods

### Peptide preparation

Aβ42 synthetic peptide (American Peptide) was solubilized in HFIP at 1 mg/ml, dried and stored at −80 °C. On the day of the experiment, the peptide was reconstituted in 2 mM NaOH to 1 mg/ml, dried and diluted in 20 mM sodium phosphate buffer, pH 8.0. The hIAPP (Phoenix Pharmaceutics) was solubilized in HFIP at 1 mg/ml, dried and stored at −80 °C. On the day of the experiment, the peptide was solubilized in 20 mM sodium phosphate buffer, pH 7.6. α-Synuclein (Bioneer) was solubilized in PBS; the transthyretin V30M mutant (Arvys Proteins) was diluted in 10 mM sodium phosphate, pH 7.6, 100 mM KCl, 1 mM EDTA.

### Heterologous expression and purification of *mt*NUCB1

Recombinant expression of NUCB1 has been described elsewhere^43^. In brief, cDNA clones for human NUCB1 corresponding to residues 31–461 and point mutations D253K, E264A, D305K, and E316A were cloned into pET28a expression vector (Amersham Biosciences) in frame with an N-terminal histidine tag (His6). His6-*mt*NUCB1 was expressed in BL21 (DE3) at 37 °C to A600 nm of 0.7 then induced with 500 μM isopropyl β-D-1-thiogalactopyranoside (IPTG, United States Biological) at 17 °C overnight. His6-*mt*NUCB1 was purified by affinity chromatography using a nickel nitrilotriacetic acid (Ni-NTA) column pre-equilibrated with buffer A (20 mM sodium phosphate, 150 mM NaCl, pH 8, 50 mM β-mercaptoethanol). The bound protein was eluted from the column by using buffer A supplemented with 500 mM imidazole. The histidine tag was cleaved by PreScission protease and both tag and protease were removed by flowing the protein solution over Ni-NTA and GST columns. *mt*NUCB1 was purified using a Superdex200 26/60 HR column equilibrated with buffer S (20 mM sodium phosphate, pH 8) to obtain homogeneously pure protein.

### CD Spectroscopy

Secondary structure measurements at 25 °C for 8 μM *mt*NUCB1 solution in 50 mM Tris-HCl, pH 8.0, 150 mM NaCl using CD were performed using an Aviv 62 A DS CD spectrophotometer. Spectra were recorded over the wavelength range of 190–250 nm at 1 nm intervals with an averaging time of 3 s using a 0.1-cm path length cell. A background spectrum was subtracted from each of the collected data sets. Each spectrum obtained was an average of 3 scans. The thermal unfolding of *mt*NUCB1 was monitored using CD at a wavelength of 222 nm, which is characteristic of an α-helix structure. The data points were averaged over 30 s for every unit increment in temperature.

### Thioflavin-T binding assay

The kinetics of aggregation of Aβ42, hIAPP, α-synuclein and the transthyretin V30M mutant was monitored, in the absence and presence of *mt*NUCB1, by using the Thio-T fluorescence assay. The peptides were diluted to desired molar concentration and 10 μM Thio-T (Fisher Scientific), with or without *mt*NUCB1. A volume of 50 μl per well (n = 4/group) was added to each well of a pre-chilled (4 °C) Corning 96 well half area black with clear flat bottom polystyrene with non-binding surface and covered with clear self-adhesive topseal. Fluorescence measurements were performed on a Flexstation II (Molecular Devices) using an excitation wavelength of 450 nm and an emission wavelength of 485 nm. Aβ42, transthyretin V30M mutant and hIAPP aggregation were tested every 10 min for up to 24 h in quiescent conditions and a constant temperature of 37 °C (Aβ42, V30M) or 25 °C (hIAPP). α-synuclein was incubated at 37 °C on a shaker and the endpoint fluorescence was measured at different time points. The obtained fluorescence measures were normalized to the relative fluorescence expressed after 30 min of incubation.

### Transmission electron microscopy

For the EM negative staining experiment (Supplementary Fig. S2), different concentrations of Aβ42 were incubated at 37 °C for 24 h and successively placed in a volume of 5 μl onto carbon film 200-mesh copper grids, rinsed with ddH2O and counterstained with 1% aqueous uranyl acetate solution. For the single immunoEM experiment (Supplementary Fig. S2), Aβ42 was incubated at 37 °C for 0, 1 h or 24 h at 10 μM monomeric concentration. Samples were incubated in solution with anti-Aβ 6E10 (BioLegend, 1:100) antibody for 20 min at room temperature, then plated on the grids in a volume of 5 μl, blocked with 3% BSA for 3 min and successively incubated with the 12 nm gold-conjugated secondary antibody (Jackson Laboratories, 1:20) for 20 min. The grids were then rinsed in buffer and stained with 1% aqueous uranyl acetate solution. For the double immunoEM experiments 10 μM Aβ42, 10 μM Aβ42 plus 10 μM *mt*NUCB1 and 10 μM *mt*NUCB1 (Fig. 3A) incubated at 37 °C for 24 h or *mt*NUCB1-Aβ42 protofibrils (Fig. 3B) were stained with the mouse anti-Aβ 6E10 (BioLegend, 1:100) antibody and the rabbit anti-NUCB1 (Aviva Systems Biology, 1:100) antibody, in solution for 20 min at room temperature. Samples were then diluted to 5 μM and placed in a volume of 5 μl onto carbon film 200-mesh copper grids for 2 min, followed by a 3 min incubation with 3% BSA. All grids were then incubated for 20 min with an anti-rabbit 12-nm gold-conjugated secondary antibody together with an anti-mouse 6 nm gold-conjugated secondary antibody (Jackson Laboratories, 1:20). The grids were then extensively rinsed in buffer and counterstained with 1% aqueous uranyl acetate solution. Samples were viewed with a JEOL JEM 1400 Plus Transmission Electron Microscope and images acquired with Gatan Digital Micrograph 1000 (a gift from Helmsley Charitable Trust).

### Kinetic analysis

Aggregation of Aβ42 was measured by Thio-T assay and for each concentration the half-time of aggregation was calculated using the AmyloFit online software (http://www.amylofit.ch.cam.ac.uk). Data were graphed in a double logarithmic plot with increasing Aβ42 concentrations where the resulting slope gives the scaling exponent. Further, rate constants of aggregation (primary nucleation, elongation and secondary nucleation) were calculated with AmyloFit and simulations were performed to determine how *mt*NUCB1 affects the global Aβ42 aggregation profile by interfering with and inhibiting microscopic aggregation event(s). First, following the preliminary steps in the AmyloFit pipeline, we chose time windows from reaction start point to plateau and normalized the values to 1. The fitted model of secondary nucleation dominant aggregation was chosen according to the guidelines published in Meisl *et al*.^35^. Successively, the model parameters [initial monomer concentration (m_0_), initial fibril number concentration (P_0_), initial fibril mass concentration (M_0_), reaction order of primary nucleation (n_c_) and reaction order of secondary nucleation (n_2_)] were set to Global constant (see Supplementary Table S1 and Supplementary Table S2 for specific values) and each time one of the rate constants was set to ‘Fit’ while the others were set to ‘Global fit’. We made sure convergence was attained by increasing the Basin Hops and observing no change in the MRE. The fitting results expressed as MRE and residuals over reaction time are analysed and shown in each of these specific fittings separately.

### Quantification of monomer and soluble aggregate content

The content of monomer Aβ42 post aggregation with or without *mt*NUCB1 was determined though SEC as previously described^60^,^61^. Specifically, 10 μM Aβ42 was incubated with varying concentrations of *mt*NUCB1 for 24 h under quiescent conditions at 37 °C. Insoluble fibril material was removed through ultracentrifugation at 55,000 × g for 60 min and the supernatant fraction was collected. Supernatant samples were injected on a calibrated Superdex 75 (1 × 30 cm) column equilibrated with 20 mM sodium phosphate pH 8.0 buffer. Eluted fractions of monomer, corresponding to 12–15 mls, were pooled and lyophilized to concentrate the peptide. The samples were resolubilized with water and the relative abundance of Aβ42 was detected by indirect ELISA with a rabbit polyclonal anti- Aβ antibody directed toward human Aβ aa 1–14 (Abcam). A portion of the original supernatant was used in a indirect ELISA for the detection of soluble aggregate material using the anti-oligomer A11 antibody (ThermoFisher Scientific).

### Size Exclusion Chromatography

The *mt*NUCB1-capped material was prepared as follows: 5 μM *mt*NUCB1 and 20 μM Aβ42 peptide were reacted under controlled conditions at 37 °C for 24 h. *mt*NUCB1 (10 μM) and hIAPP (33 μM) peptide were incubated at 37 °C for 3 h while stirred. *mt*NUCB1 (50 μM) and α-synuclein (100 μM) were co-incubated at 37 °C for 24 h on a shaker (1500 RPM). *mt*NUCB1 (20 μM) and V30M (20 μM) were co-incubated at 37 °C for 24 h on a shaker (1500 RPM). Capped-protofibril containing solutions were then applied to a Superdex200 26/60 PG SEC column (GE Healthcare, Piscataway, NJ) equilibrated with buffer (for *mt*NUCB1-Aβ42: 20 mM sodium phosphate, pH 8.0, 150 mM NaCl; for *mt*NUCB-hIAPP: 20 mM sodium phosphate, pH 7.6, 150 mM NaCl; for *mt*NUCB1-α-synuclein: PBS; for *mt*NUCB1-V30M: 10 mM sodium phosphate, pH 7.6, 100 mM KCl, 1 mM EDTA). For each sample, the main peak was collected for subsequent experiments.

### Cell Toxicity Assay

PC12 cells (25000 cells/well) were pre-incubated overnight in 96-well plates. The cells were treated with Aβ42 in the presence or absence of *mt*NUCB1 for 24 h. MTT cell proliferation assays (Roche) were performed by treating PC12 cells with MTT labelling solution for 4 h followed by an overnight cell solubilisation. Purple formazan crystals were detected with a Spectra Max 250 (Molecular Devices).

### ELISA Assay

Indirect ELISA assays were performed using Maxisorp 96-well plates (NUNC) coated for 2 h at room temperature with 50 μl/well of sample in coating buffer (0.2 M carbonate buffer, pH 9.5). After rinsing three times with 75 μl Protein Free Blocking Buffer (Pierce) the wells were blocked with Protein Free Blocking Buffer for 2 h. The wells were then incubated overnight at 4 °C with 50 μl of detection antibody prepared in blocking buffer. After three washes with TBST, the wells were incubated for 2 h with 50 μl HRP-conjugated reaction antibody. After three washes, 100 μl of freshly made Amplex UltraRed (Invitrogen) substrate solution (5 μM Amplex UltraRed in 50 mM sodium citrate, pH 6.0 with the addition of H_2_O_2_) was added. After 45 min incubation at room temperature in the dark, HRP activity was detected by measuring fluorescence with a microplate reader set for excitation in the range of 530–560 nm and emission-detection at 590 nm.

### Atomic Force Microscopy

Imaging was performed in air using a combination of the Cypher ES and the MFP-3D-BIO AFMs (Asylum Research, Goleta, CA). All images were acquired in tapping mode using Olympus AC240TS-R3 probe (Asylum Research, Goleta CA). Samples were prepared in stock solutions, diluted to the desired working concentration, and immediately plated in a volume of 40 μl on freshly cleaved mica (SPI). After incubation (between 10 sec and 4 min, depending on the sample), the samples were washed under a gentle stream of 10 ml molecular biology grade H2O (Fisher BP2819–1) before being blown dry with N2 gas. The samples were immediately placed under the AFM stage and high-resolution images (1 μm x 1 μm, 512 × 512 pixels) were acquired.

Raw data were exported into 8-bit grayscale tiff images using the Asylum Research’s Igor Pro software and then imported into FIJI/ImageJ (NIH) for volume quantification using a custom written FIJI code. A height threshold of 0.39 nm–1.52 nm was set for each protofibril samples, as well as a pixel area threshold, to exclude noise from the image. For each protofibrillar species, 1000–10000 individual particles were analyzed. Volume of each structure was calculated using the formula


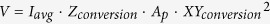


where *I*_*avg*_ is the average intensity, *Z*_*conversion*_ is the conversion of one gray scale unit of intensity into height in nanometers, *XY*_*conversion*_ is the pixel to nanometer conversion for the image in xy, and *A*_*p*_ is the area of particles in pixels. The top 10% of pixels was used to determine the height of each protofibril/protein structure, and a Feret’s diameter measurement was used to get the protofibrillar length. Each segmented structure was then cropped into its own individual image, a bicubic interpolation was applied, and the image was saved to create montages of individual protofibrils/proteins from the AFM data. A volume histogram was created and particles were chosen in a range around the volume with the highest frequency of events, depending on the heterogeneity of the sample (*i.e*., more narrow distribution, smaller range, and wider distribution, larger range). Two more custom-made FIJI macros were developed to create appropriate scale bars for the montages and to put the images into 7 × 7 grid with the LUT imported from the Asylum Research Igor Pro software (the Z-scale bar was acquired from raw data image). Volume analysis was performed with Graphpad Prism and data were plotted as a probability density function.

### Dynamic Light Scattering

The Aβ42-*mt*NUCB1 complex purified with SEC and the *mt*NUCB1 only sample were diluted to 1 μM and plated in a volume of 60 μl per well in 384-well plates (Greiner Bio-One). The intensity of the light scattered by the particles in solution as well their hydrodynamic radius (nm) was measured by Wyatt DynaPro Plate Reader II (DWB 208) and analyzed by DYNAMICS software. Each well was subjected to 10 acquisitions, 10 s each. Kernel density estimates were made in Python 2.7 using the scipy.stats.gaussian_kde module and the Silverman method for determining bandwidth.

## Additional Information

**How to cite this article:** Bonito-Oliva, A. *et al*. Nucleobindin 1 binds to multiple types of pre-fibrillar amyloid and inhibits fibrillization. *Sci. Rep.* **7**, 42880; doi: 10.1038/srep42880 (2017).

**Publisher's note:** Springer Nature remains neutral with regard to jurisdictional claims in published maps and institutional affiliations.

## Supplementary Material

Supplemental Figures
