# Supplementary material for: Nucleobindin 1 binds to multiple types of pre-fibrillar amyloid and inhibits fibrillization
Source: Sci Rep. 2017 Feb 21;7:42880. doi: 10.1038/srep42880 (PMC5318909; doi:10.1038/srep42880)
Supplement: Supplemental Figures [file srep42880-s1.pdf]

Supplementary Material to:

Nucleobindin 1 binds to multiple types of pre-fibrillar amyloid and inhibits fibrillization

Alessandra Bonito-Oliva[1], Shahar Barbash[1], Thomas P. Sakmar[1,2#] and W Vallen Graham[1#]

[1]Laboratory of Chemical Biology & Signal Transduction, The Rockefeller University, New York, NY 10065, USA. [2]Department of Neurobiology, Care Sciences and Society, Center for Alzheimer Research, Division of Neurogeriatrics, Karolinska Institutet, 141 57 Huddinge, Sweden

Correspondence and requests for materials should be addressed to T.P.S. (email: sakmar@rockefeller.edu) or to W.V.G. (email: vgraham@rockefeller.edu).

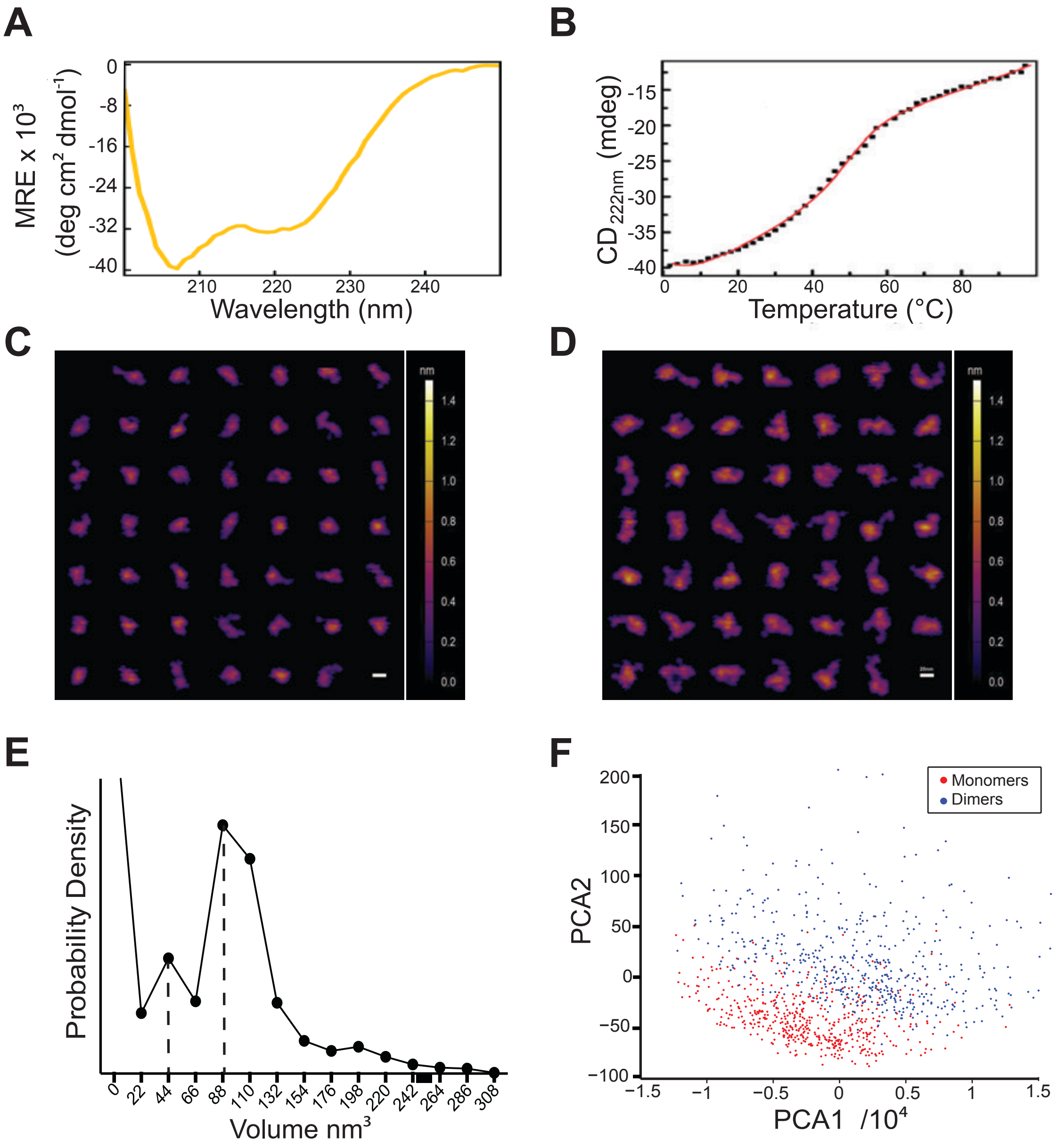

Supplementary Fig. S1. Characterization of *mt*NUCB1. **A** Circular Dichroism (CD) spectroscopy of (8µM) *mt*NUCB1 shows a spectrum (Mean Residue Ellipticity, MRE) characteristic of a fully folded protein with significant helical secondary structure. **B** Thermal unfolding of (8µM) *mt*NUCB1 monitored by recording CD signal at 222 nm at a heating rate of 1 °C / min indicates an apparent Tm of 48.7 °C. **C** Composite of representative *mt*NUCB1 monomers and **D** dimers imaged by AFM. Integrated xy Scale Bar = 20nm; colorimetric scale bar indicates the height of the particles. **E** Volume analysis of *mt*NUCB1 was performed with a bin size = 22 and expressed as probability density for volume (nm³). It reveals a predominant peak at 88 nm³ and a smaller peak at ~44 nm³, as indicated by the dashed lines. **F** Principal component analysis (PCA) performed on the amplitude of the 12 most significant frequencies obtained by Fourier transform shows the different shape distribution of *mt*NUCB1 monomers and dimers.

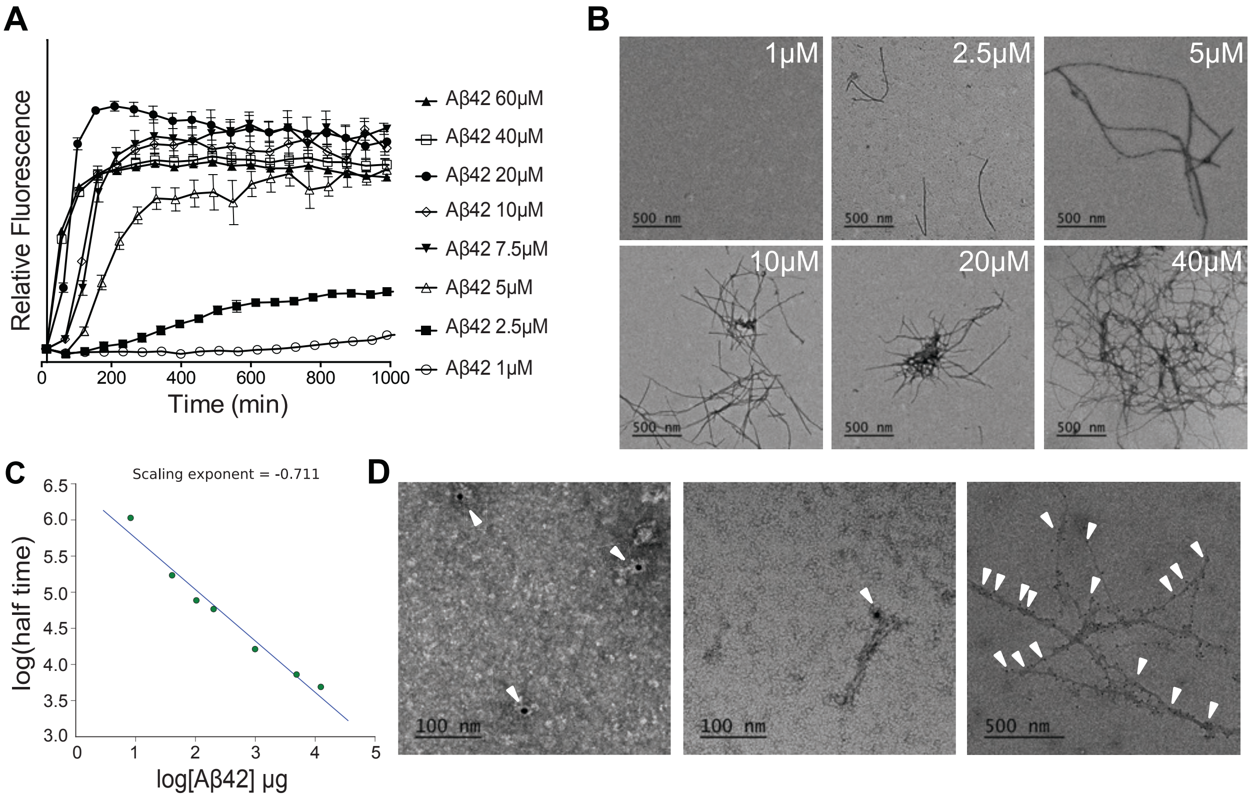

Supplementary Fig. S2. Aggregation kinetics of Aβ42. A Aggregation kinetics of Aβ42 measured by Thio-T assay, at 37 °C in quiescent conditions, over 24 h. Aβ42 was diluted to various monomeric concentrations and incubated together with 10μM Thio-T. Excitation wavelength was 450 nm and emission wavelength was 485 nm. **B** Representative Electron Microscopy (EM) images of Aβ42 incubated at 37 °C at different monomeric concentrations for 24 h. Scale bar = 500 nm. **C** The half-time of Aβ42 aggregation was calculated with AmyloFit and graphed together with increasing Aβ42 concentrations in a double logarithmic plot. The data (green circles) results in a straight slope (blue line) that gives a scaling exponent = -0.771 indicative of a secondary nucleation dominant mechanism of aggregation. **D** Representative immunoEM images of Aβ42 incubated at 37 °C for 0, 1 h or 24 h at 10μM monomeric concentration. Samples were incubated with mouse anti-Aβ 6E10 antibody and 12 nm gold-conjugated secondary antibody. Panels are representative images of early aggregates (right, 100 nm scale bar), protofibrils (centre, scale bar = 100 nm) and fibrils (left, scale bar = 500 nm). Arrowheads indicating 12 nm gold particles (Aβ).

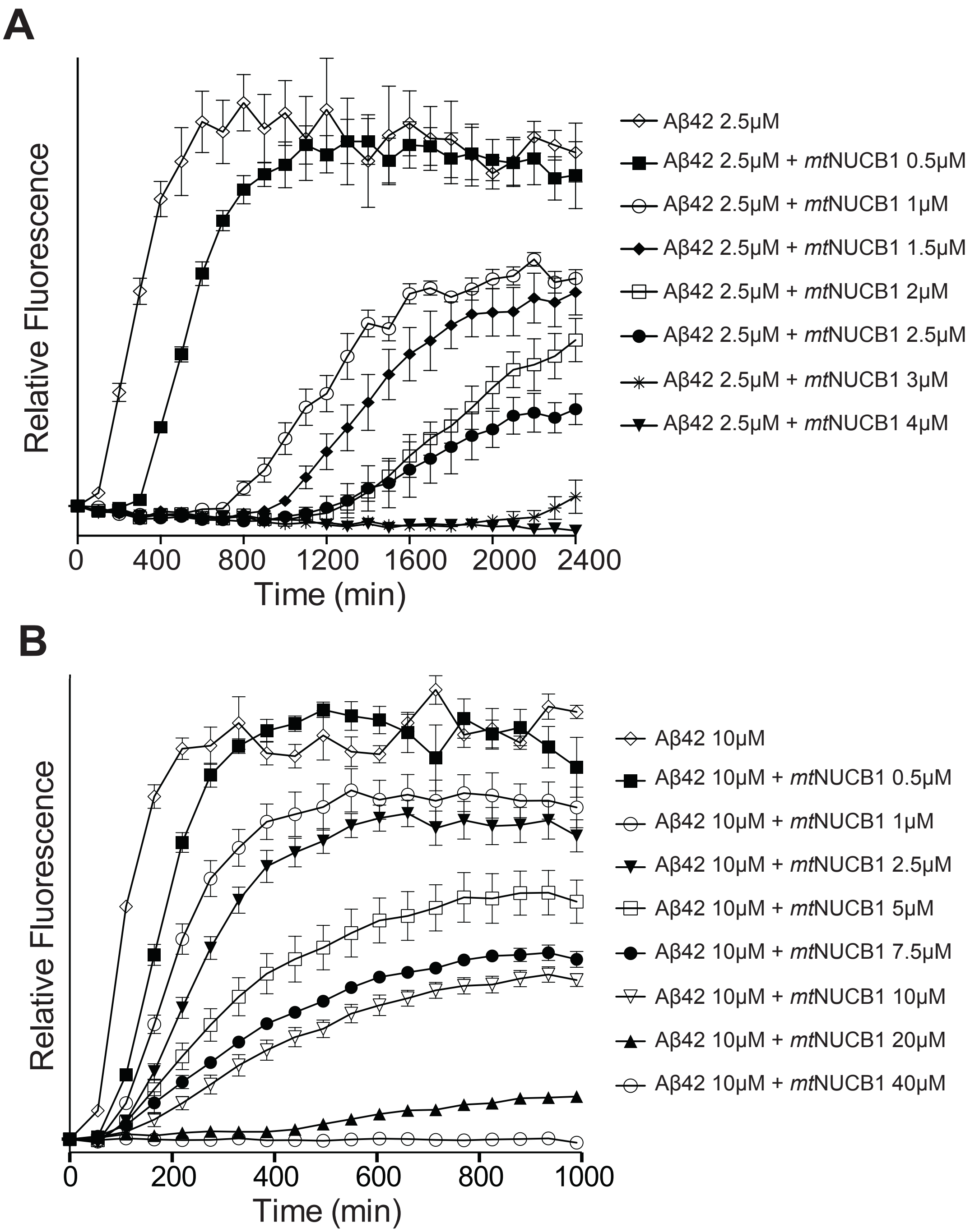

Supplementary Fig. S3. mtNUCB1 effect on Aβ42 monomeric content. A Dilution dependent effect of *mt*NUCB1 on 2.5μM and **B** 10μM Aβ42 aggregation, measured by a Thio-T assay, at 37 °C in quiescent conditions, over 40 h and 24 h, respectively. The *mt*NUCB1 was diluted to various concentrations and incubated together with 2.5μM or 10μM Aβ42 and 10μM Thio-T.

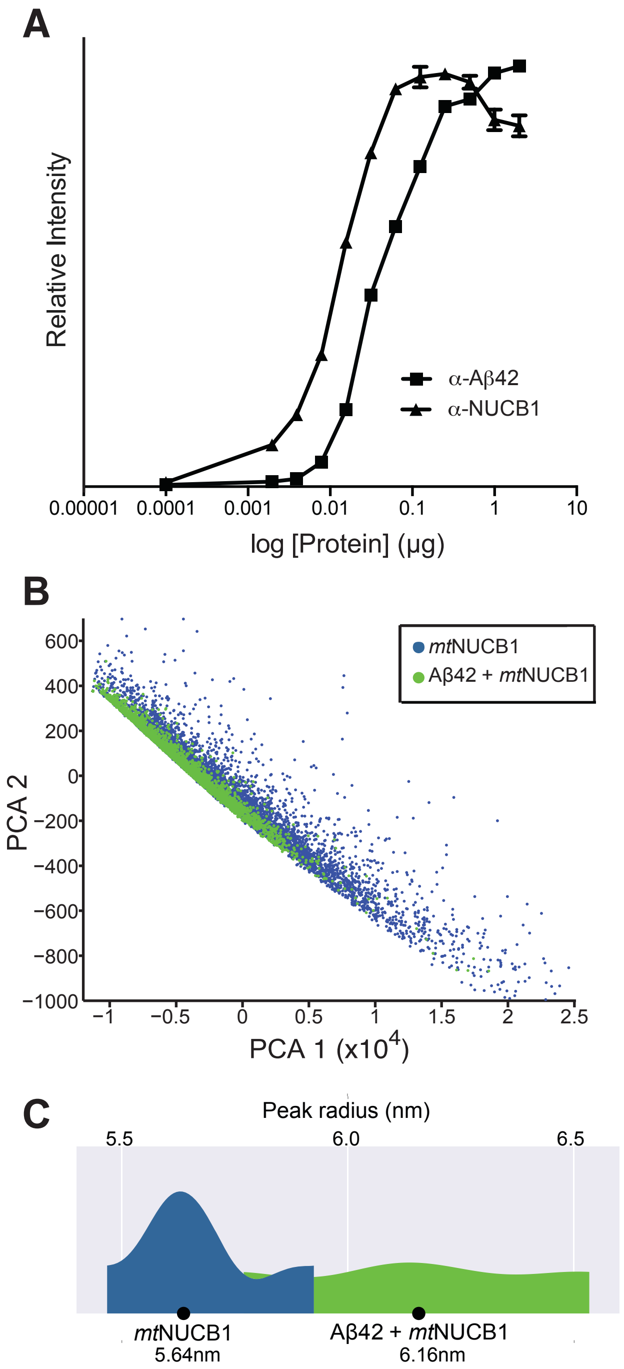

Supplementary Fig. S4. Characterization of *mt*NUCB1-Aβ42 protofibrils. *mt*NUCB1-Aβ42 protofibrils were purified with SEC and characterized by different assays. **A** Direct ELISA shows that both Aβ42 and *mt*NUCB1 are detected in the sample by using anti-Aβ 6E10 and anti-NUCB1 antibodies. **B** The *mt*NUCB1-Aβ42 protofibrils were imaged by AFM and the principal component analysis (PCA) was performed on the amplitude of the 12 most significant frequencies obtained by Fourier transform. The graph shows that the *mt*NUCB1-Aβ42 complex has a less spread shape distribution compared to *mt*NUCB1 (monomers + dimers). **C** DLS analysis shows the hydrodynamic peak radius of *mt*NUCB1-Aβ42 protofibrils (6.16 nm) and *mt*NUCB1 (5.6 nm).

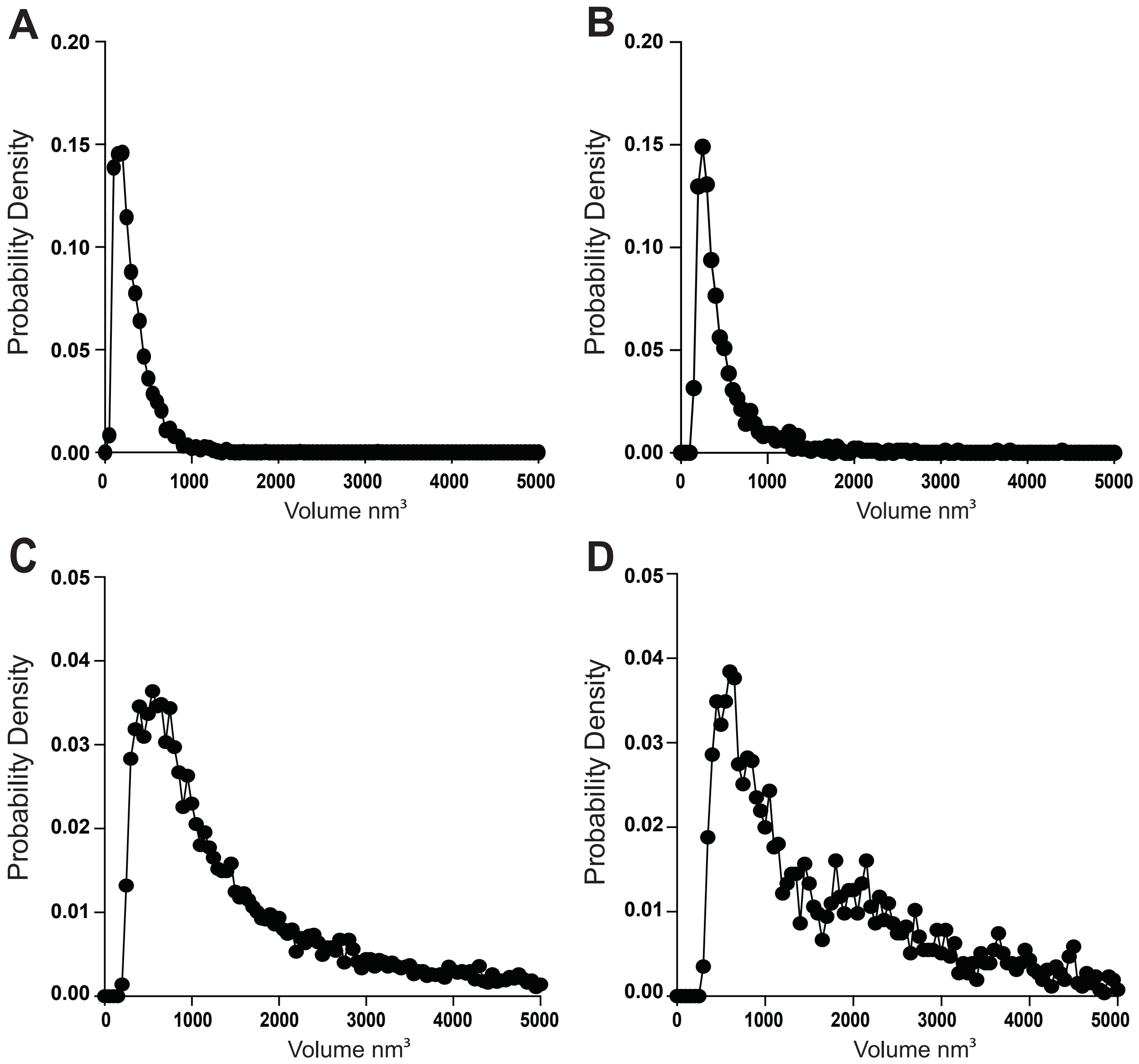

Supplementary Fig. S5. *mt*NUCB1 binding to different amyloidogenic pre-fibrillar species generates protofibrils of different sizes. *mt*NUCB1 was incubated together with **A** Aβ 42, **B** hIAPP, **C** α-synuclein, or **D** the transthyretin V30M mutant and the obtained protofibrils were purified by SEC and imaged by AFM. The volumetric analysis performed on the images is displayed as discrete probability density functions. It indicates a size peak of 250 nm^3, 550 nm^3, 600 nm^3 and 200 nm^3 for *mt*NUCB1-Aβ42, *mt*NUCB1-hIAPP, *mt*NUCB1-α-synuclein and *mt*NUCB1-V30M protofibrils, respectively, as indicated by the dashed lines. The area under the curve represents the size distribution and is an indication of volume heterogeneity.

Fitting for Primary nucleation								
Dataset	P_0	m_0	n_c	k_n	k_2	k_p	M_0	n_2
Units	μM	μM	unitless	μM ^{-n_c+1} min^{-1}	μM^{-n_2} min^{-1}	μM ^{-1} min^{-1}	μM	unitless
Aβ42 10μM	0	1.00E-05	2	16.75817	557988.1	73313.81	0	2
Aβ42 10μM + *mt*NUCB1 0.5μM	0	1.00E-05	2	6.890221	557988.1	73313.81	0	2
Aβ42 10μM + *mt*NUCB1 1μM	0	1.00E-05	2	4.188004	557988.1	73313.81	0	2
Aβ42 10μM + *mt*NUCB1 2.5μM	0	1.00E-05	2	2.493036	557988.1	73313.81	0	2
Aβ42 10μM + *mt*NUCB1 5μM	0	1.00E-05	2	1.212494	557988.1	73313.81	0	2
Aβ42 10μM + *mt*NUCB1 7.5μM	0	1.00E-05	2	0.880386	557988.1	73313.81	0	2
Aβ42 10μM + *mt*NUCB1 10μM	0	1.00E-05	2	0.592188	557988.1	73313.81	0	2
**FItting for Elongation**								
Dataset	P_0	m_0	n_c	k_n	k_2	k_p	M_0	n_2
Units	μM	μM	unitless	μM ^{-n_c+1} min^{-1}	μM^{-n_2} min^{-1}	μM ^{-1} min^{-1}	μM	unitless
Aβ42 10μM	0	1.00E-05	2	33.55505	29779315	15541.79	0	2
Aβ42 10μM + *mt*NUCB1 0.5μM	0	1.00E-05	2	33.55505	29779315	6824.286	0	2
Aβ42 10μM + *mt*NUCB1 1μM	0	1.00E-05	2	33.55505	29779315	4697.141	0	2
Aβ42 10μM + *mt*NUCB1 2.5μM	0	1.00E-05	2	33.55505	29779315	3214.001	0	2
Aβ42 10μM + *mt*NUCB1 5μM	0	1.00E-05	2	33.55505	29779315	1966.506	0	2
Aβ42 10μM + *mt*NUCB1 7.5μM	0	1.00E-05	2	33.55505	29779315	1610.116	0	2
Aβ42 10μM + *mt*NUCB1 10μM	0	1.00E-05	2	33.55505	29779315	1309.578	0	2
**Fitting for Secondary nucleation**								
Dataset	P_0	m_0	n_c	k_n	k_2	k_p	M_0	n_2
Units	μM	μM	unitless	μM ^{-n_c+1} min^{-1}	μM^{-n_2} min^{-1}	μM ^{-1} min^{-1}	μM	unitless
Aβ42 10μM	0	1.00E-05	2	3.031234	59889263	27150.8	0	2
Aβ42 10μM + *mt*NUCB1 0.5μM	0	1.00E-05	2	3.031234	16109753	27150.8	0	2
Aβ42 10μM + *mt*NUCB1 1μM	0	1.00E-05	2	3.031234	8993118	27150.8	0	2
Aβ42 10μM + *mt*NUCB1 2.5μM	0	1.00E-05	2	3.031234	4675770	27150.8	0	2
Aβ42 10μM + *mt*NUCB1 5μM	0	1.00E-05	2	3.031234	1575275	27150.8	0	2
Aβ42 10μM + *mt*NUCB1 7.5μM	0	1.00E-05	2	3.031234	943518.8	27150.8	0	2
Aβ42 10μM + *mt*NUCB1 10μM	0	1.00E-05	2	3.031234	539043.7	27150.8	0	2

Supplementary Table S1. Kinetic analysis of 2.5μM Aβ42. The kinetic data of 2.5μM Aβ42 aggregation were analysed with AmyloFit to perform individual simulations. All the parameters [initial monomer concentration (m_0), initial fibril number concentration (P_0), initial fibril mass concentration (M_0), reaction order of primary nucleation (n_c), and reaction order of secondary nucleation (n_2)] were set to Global constant and each time one of the rate constants (primary nucleation, elongation, or secondary nucleation) was set to 'Fit' while the others were set to 'Global fit'. Each table represents a single fit where P_0, m_0, n_c, M_0 and n_2 were kept as global constants and only one of the rate constants was individually fitted while the other two were globally fitted.

**Fitting for Primary nucleation**								
Dataset	P_0	m_0	n_c	k_n	k_2	k_p	M_0	n_2
Units	μM	μM	unitless	μM ^{-n_c+1} min^{-1}	μM^{-n_2} min^{-1}	μM ^{-1} min^{-1}	μM	unitless
Aβ42 2.5μM	0	2.50E-06	2	106.7625356	75428840.97	26049.52202	0	2
Aβ42 2.5μM + *mt*NUCB1 0.5μM	0	2.50E-06	2	8.236769202	75428840.97	26049.52202	0	2
Aβ42 2.5μM + *mt*NUCB1 1μM	0	2.50E-06	2	0.04926456	75428840.97	26049.52202	0	2
Aβ42 2.5μM + *mt*NUCB1 1.5μM	0	2.50E-06	2	0.007274007	75428840.97	26049.52202	0	2
Aβ42 2.5μM + *mt*NUCB1 2.5μM	0	2.50E-06	2	0.000817747	75428840.97	26049.52202	0	2
**FItting for Elongation**								
Dataset	P_0	m_0	n_c	k_n	k_2	k_p	M_0	n_2
Units	μM	μM	unitless	μM ^{-n_c+1} min^{-1}	μM^{-n_2} min^{-1}	μM ^{-1} min^{-1}	μM	unitless
Aβ42 2.5μM	0	2.50E-06	2	1.325076673	2514054993	15428.89832	0	2
Aβ42 2.5μM + *mt*NUCB1 0.5μM	0	2.50E-06	2	1.325076673	2514054993	3972.711546	0	2
Aβ42 2.5μM + *mt*NUCB1 1μM	0	2.50E-06	2	1.325076673	2514054993	817.2005946	0	2
Aβ42 2.5μM + *mt*NUCB1 1.5μM	0	2.50E-06	2	1.325076673	2514054993	559.5560022	0	2
Aβ42 2.5μM + *mt*NUCB1 2.5μM	0	2.50E-06	2	1.325076673	2514054993	392.1819611	0	2
**Fitting for Secondary nucleation**								
Dataset	P_0	m_0	n_c	k_n	k_2	k_p	M_0	n_2
Units	μM	μM	unitless	μM ^{-n_c+1} min^{-1}	μM^{-n_2} min^{-1}	μM ^{-1} min^{-1}	μM	unitless
Aβ42 2.5μM	0	2.50E-06	2	1.109191521	1.50E+11	536.7616984	0	2
Aβ42 2.5μM + *mt*NUCB1 0.5μM	0	2.50E-06	2	1.109191521	30495522889	536.7616984	0	2
Aβ42 2.5μM + *mt*NUCB1 1μM	0	2.50E-06	2	1.109191521	4444849031	536.7616984	0	2
Aβ42 2.5μM + *mt*NUCB1 1.5μM	0	2.50E-06	2	1.109191521	2777244362	536.7616984	0	2
Aβ42 2.5μM + *mt*NUCB1 2.5μM	0	2.50E-06	2	1.109191521	1777569102	536.7616984	0	2

Supplementary Table S2. Kinetic analysis of 10μM Aβ42. The kinetic data of 10μM Aβ42 aggregation were analysed with AmyloFit to perform individual simulations. All the parameters [initial monomer concentration (m_0), initial fibril number concentration (P_0), initial fibril mass concentration (M_0), reaction order of primary nucleation (n_c), and reaction order of secondary nucleation (n_2)] were set to Global constant and each time one of the rate constants (primary nucleation, elongation, or secondary nucleation) was set to 'Fit' while the others were set to 'Global fit'. Each table represents a single fit where P_0, m_0, n_c, M_0 and n_2 were kept as global constants and only one of the rate constants was individually fitted while the other two were globally fitted.
